# Summer Camp Health Initiative: An Overview of Injury and Illness in Two Canadian Summer Camps

**DOI:** 10.7759/cureus.2905

**Published:** 2018-07-01

**Authors:** Adam Handler, Mattan Lustgarten, Arielle Zahavi, Daniel Freedman, Les Rosoph, Katrina F Hurley

**Affiliations:** 1 University of Toronto, Toronto Western Hospital, Torotno, CAN; 2 Faculty of Medicine, University of Toronto, Toronto, CAN; 3 Medicine, Technion American Medical School, Haifax, ISR; 4 Dermatology, Northern Ontario School of Medicine, North Bay, CAN; 5 Emergency Department, IWK Health Centre, Halifax, CAN

**Keywords:** summer camp, residential camp, illness, injury, infirmary, rural medicine, injury prevention

## Abstract

Introduction: Summer camp is an important part of the lives of millions of youth worldwide. Injuries and illnesses at general residential camps have not been quantified in a Canadian setting. The objective of this study was to examine the incidence of injuries and illnesses that present to camp health centres at two Canadian residential summer camps.

Method: This prospective cross-sectional study examined the incidence of new-onset injuries and illnesses that presented to camp infirmaries and circumstances surrounding their occurrence. Data collection forms were completed by trained infirmary staff during each camper’s presentation to the infirmary at two general residential camps in Canada in the summers of 2015 and 2016.

Results: There were 1872 infirmary presentations, resulting in a frequency of 52.6 presentations per 1000 camp days (CD). The incidence of illness was 34.8 per 1000 CD and the incidence of injury was 17.9 per 1000 CD. Communicable disease was the most common diagnosis (15.2/1000 CD), most often an upper respiratory tract infection. The most common symptoms upon presentation were sore throat (14.1/1000 CD), headache (9.9/1000 CD), runny nose/congestion (6.2/1000 CD), cough (6.0/1000 CD) and nausea and vomiting (4.8/1000 CD). The most common injuries were cuts/lacerations/bruises (4.9/1000 CD), followed by muscle/tendon injury (4.9/1000 CD). The most frequent cause of injuries was participation in sports (3.9/1000 CD) and sports fields and courts were the most frequent location for injuries to occur (2.7/1000 CD). Females accounted for 52.8% of infirmary presentations. Senior campers (ages 12-16 years) presented most frequently (43.4%), followed by junior campers (ages 6-11 years; 38.1%) and staff (age ≥17 years; 18.0%). When age-specific CDs were calculated, junior campers had the highest frequency of infirmary presentations relative to their time spent at camp (79.7/1000 CD). Fifty people (1.4/1000 CD) were sent to a hospital for further assessment.

Conclusion: Injuries and illnesses presenting for infirmary care in summer camp are generally minor in nature. Canadian data compares similarly to United States (US) studies. Future studies should focus on interventions to reduce these injuries and illnesses, particularly communicable illnesses.

## Introduction

Summer camp is an important part of the lives and development of millions of youth around the world. In Ontario alone, there are over 450 summer camps associated with the Ontario Camps Association [1]. Many of these camps are residential (overnight) and consist of intimate, rural environments that provide campers and staff with cohabitation and routines that differ significantly from typical urban lifestyles. Close living conditions, together with an unfamiliar terrain, supervision, and activities pose a risk of injury and illness. Camps are tasked with providing a safe experience to minimize the potential negative impact of these events on camper well-being [2].

Research regarding injuries and illnesses in general residential camps has not yet been conducted in a Canadian setting. Within the existing literature in American camp settings, there is considerable variability in definitions of injury and illness, study methodology, and outcomes. Events are reported through methods such as retrospective treatment logs, phone/internet reporting, and weekly reports. Definitions of injury/illness vary from including all infirmary presentations to including only those incidents that cause disruption to the individual’s camp routine. Additionally, there are limited levels of on-site monitoring of data collection practices [2-5].

Prior research has described rates of injuries and illnesses using the “per 1000 camp days (CD)” metric, with one CD representing one staff member or camper at camp for one day. Using this metric, rates range from 0.47-0.50 injuries per 1000 CD and 0.83-1.38 illnesses per 1000 CD [2-4]. The most common injuries reported have been sprains/strains, cuts, abrasions, splinters, bruises, and fractures [2-4]. Lower extremities were more often injured, and campers were more prone to injury than staff [2]. Illnesses exceed injuries, with the most frequent presentations including maladies of the respiratory and gastrointestinal tracts [2-5]. Overall, the likelihood of sustaining a serious injury or illness at camp is low [2-5].

Human factors have been shown to play a larger role than the physical environment in the occurrence of injuries [6]. Garst et al. identified major risks for injury and illness including arriving at camp ill, slipping/tripping/falling, inadequate or improper use of protective equipment, and supervision [2]. Additionally, while Goldlust et al. found no association between “significant injury” (at least 24 hours of restriction from camp activities) and age, sex, time of day, level of supervision, location, or camper-to-staff ratio; other studies have shown associations between injuries and one or more of gender, age, or time of day [5,7-8].

The objective of this study was to examine the incidence of injuries and illnesses that present to camp health centres amongst individuals attending two Canadian residential summer camps.

## Materials and methods

Design

This is a prospective study involving a convenience sample of two Canadian residential camp cohorts. Both camp directors expressed written informed consent to permit data collection at the camp infirmaries, through use of paper-based data collection forms. Ethics approval was obtained from the Queen’s University Health Sciences Research Ethics Board (file number 6015206).

Setting

Data were collected at two Canadian residential summer camp infirmaries during the summers of 2015 and 2016. The camps spanned six to eight weeks with a portion of campers staying for just the first 10 days or three weeks. Attendees remained on site for the duration of the session, with occasional short field trips to surrounding cities or towns. The camps offered a wide range of activities such as swimming, boating, water-skiing, arts and crafts, dancing, sports, and camping. Each camp had an infirmary staffed with nursing students and trained camp personnel, and one camp had a full-time on-site physician. The nearest hospital was located nine kilometres from one of the camps and 34 kilometres from the other camp.

Participants

The sample consisted of all registered campers and employed staff (counsellors, activity specialists, and administrative personnel) at both participating summer camps. There were 977 camp residents over two years (470 in year one and 507 in year two) constituting 35578 CDs, a measure that incorporates both population size and time spent at camp by each individual. It specifically reflects the sum of the daily population of campers and staff at each camp, on each day of the week throughout the camp session [2,7]; see Appendix I.

Exclusion criteria consisted of the following: presentations determined by the physician and infirmary staff to be highly specific so as to potentially identify the presenting individual; presentations regarding mental health; and presentations to the infirmary for prescribed daily medications.

Variables

The primary outcome was the incidence of injury and illness that presented to camp infirmaries at residential summer camps using the CD metric. The secondary outcomes were the circumstances associated with the injuries/illnesses including demographic variables, the context surrounding the event (mechanism, time of day, week, location, activity, supervision and protective equipment), and the need to transfer the individual for further evaluation at the local hospital.

Measures

Data collection forms were completed by camp infirmary staff and volunteers. The data collection form was modelled after the Canadian Hospitals Injury Reporting and Prevention Program (CHIRPP) form, which is used to collect reliable and valid data on demographics, injury narrative, and the nature and location of injuries experienced by individuals visiting participating Emergency Departments [9-10]. We modified this data collection tool to include variables relevant to camp settings: specific locations and activities at camp (e.g., dining hall, sports, waterfront), common camp illnesses, supervision, hospitalization, and management. Data collection forms did not contain any identifying information. Feedback from structured meetings with camp physicians, nurses, and camp staff resulted in minor amendments to the data collection forms for year two of the study. These modifications simplified data collection without compromising the primary or secondary outcomes and added a coded identifier to track repeated infirmary visits by the same individual.

Data on injury and illness were included if they met either of the following two requirements: (1) camper/staff sought or was brought to the infirmary for medical attention, or (2) any situation outside of the infirmary that required attention from the infirmary staff. Training was provided during the pre-camp session to all infirmary staff and volunteers involved in data collection to maximize inter-observer reliability. Principal investigators provided on-site monitoring throughout the summer. Forms were kept in the camp infirmary in a secure cabinet and collected at the end of the camp sessions for data entry into a password-protected, encrypted electronic offline database.

Statistical methods

We used descriptive statistics to describe this cohort of summer camp participants. We calculated the incidence of injury and illness using the CD metric. Participants were categorized as “junior campers” (aged 6-11 years), “senior campers” (aged 12-16 years), and “staff” (age ≥17 years). Across all variables, missing data were analyzed as a separate “unknown” category.

Bias

The data collection tool was pilot tested with a medical doctor and the principal investigators by independently completing the data form for 15 predetermined presentations. Data discrepancies were addressed and corrected. Infirmary staff and volunteers were trained on how to complete the data collection form. The on-site principal investigators were able to address concerns as they arose and provided staff encouragement. The staff completing each data collection form were required to sign the form [11].

## Results

This residential camp cohort included 701 campers and 276 staff. Baseline camp resident demographics are outlined in Table 1.

**Table 1:** Demographic characteristics of camp residents

		2015	2016	Total	% Total
Camp Days		17052	18526	35578	--
Gender					
	M	246	256	502	51.4%
	F	224	251	475	48.6%
Age Group					
	Jr Camper (6-11 years)	148	150	298	30.5%
	Sr Camper (12-16 years)	181	222	403	41.2%
	Staff (≥17 years)	141	135	276	28.2%
Total		470	507	977	100%

**Figure 1:**
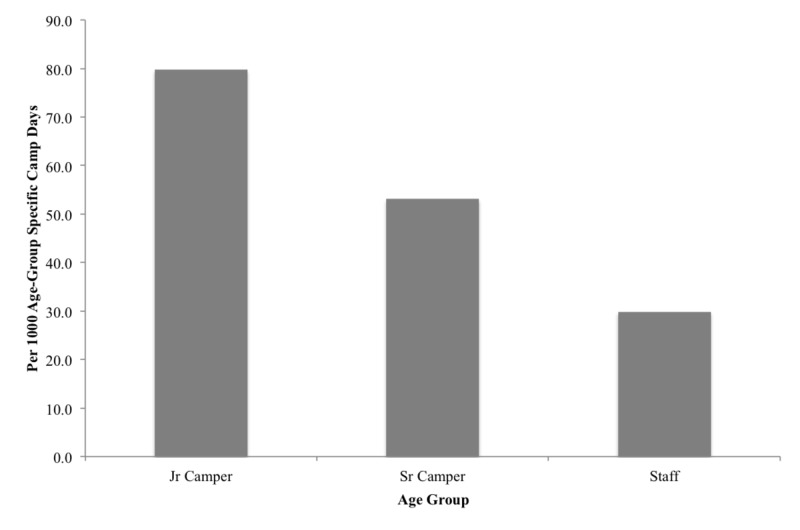
Age-group specific incidence of infirmary presentations per 1000 camp days Jr Camper: ages 6-11 years Sr Camper: ages 12-16 years Staff: ≥17 years

Over the two summers, there were 1872 infirmary presentations (52.6 presentations per 1000 CD) divided into an injury incidence of 17.9 per 1000 CD and an illness incidence of 34.8 per 1000 CD. Infirmary visitors were more often female (52.8%) and, with respect to age, senior campers visited the infirmary most frequently (43.4%) (Table 2). When frequencies were calculated according to “age-group specific camp days”, junior campers had the highest rate of presentations at 79.7 per 1000 CD, compared to 53.1 per 1000 CD for senior campers and 29.8 per 1000 CD for staff (Figure 1). The highest frequency of presentations occurred in week two of camp (28.5%), followed by week one (21.0%) and week three (19.1%) (Table 2). More infirmary presentations occurred in the morning (42.0%) and evening/night (37.6%) (Table 2). Over the two summers, 50 people (1.4 per 1000 CD) were sent to hospital for further assessment.

**Table 2:** Characteristics of infirmary presentations /1000 CD = presentations per 1000 camp days Morning = wake up-lunch (0600-1200) Afternoon = lunch-dinner (1201-1800) Evening/night = dinner-wake up (1801-0559)

		Frequency (%)	/1000 CD
Total Presentations (n)		1872 (100)	52.6
Presentation Type			
	Illness	1238 (66.1)	34.8
	Injury	632 (33.8)	17.8
	Unknown	2 (0.1)	0.1
Time At Presentation			
	Morning	787 (42.0)	22.1
	Afternoon	346 (18.5)	9.7
	Evening/Night	704 (37.6)	19.8
	Unknown	35 (1.9)	1.0
Week of Presentation			
	1	393 (21.0)	11.0
	2	533 (28.5)	15.0
	3	358 (19.1)	10.1
	4	229 (12.2)	6.4
	5	175 (9.3)	4.9
	6	154 (8.2)	4.3
	Unknown	30 (1.6)	0.8
Gender			
	F	988 (52.8)	27.8
	M	882 (47.1)	24.8
	Unknown	2 (0.1)	
Age Group			
	Jr Camper (6-11 years)	714 (38.1)	20.1
	Sr Camper (12-16 years)	813 (43.4)	22.9
	Staff (≥17 years)	337 (18.0)	9.5
	Unknown	8 (0.4)	0.2

**Figure 2:**
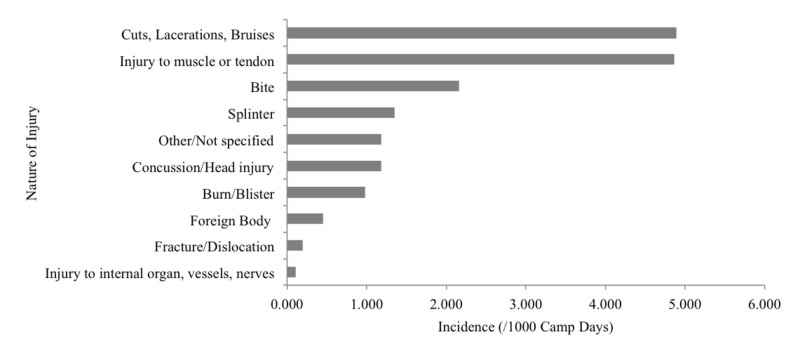
Nature of injuries per 1000 camp days Note: "Foreign Body" excludes splinter. "Injury to muscle or tendon" includes sprains and strains.

The most common injuries were cuts/lacerations/bruises (4.9 per 1000 CD), followed by muscle/tendon injury (4.9 per 1000 CD), bug bites (2.2 per 1000 CD), and splinter/sliver (1.4 per 1000 CD) (Figure 2).

**Figure 3:**
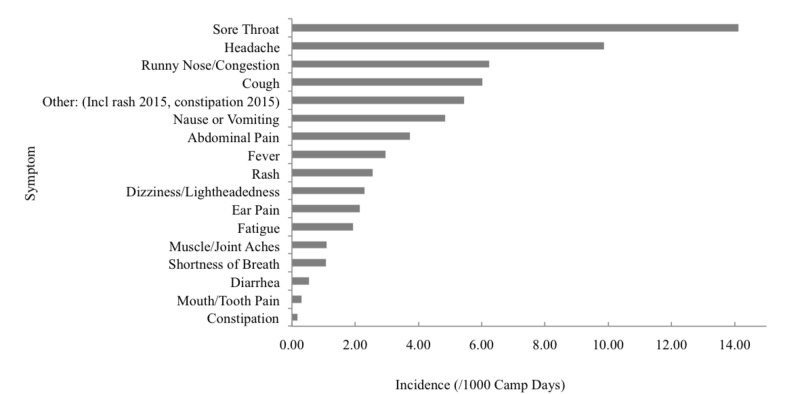
Incidence of symptoms upon illness presentation per 1000 camp days

The incidence of communicable/infectious disease was 15.2 per 1000 CD, with the most common symptoms including sore throat (14.1 per 1000 CD), headache (9.9 per 1000 CD), runny nose/congestion (6.2 per 1000 CD), cough (6.0 per 1000 CD) and nausea and vomiting (4.8 per 1000 CD) (Figure 3). 

**Figure 4:**
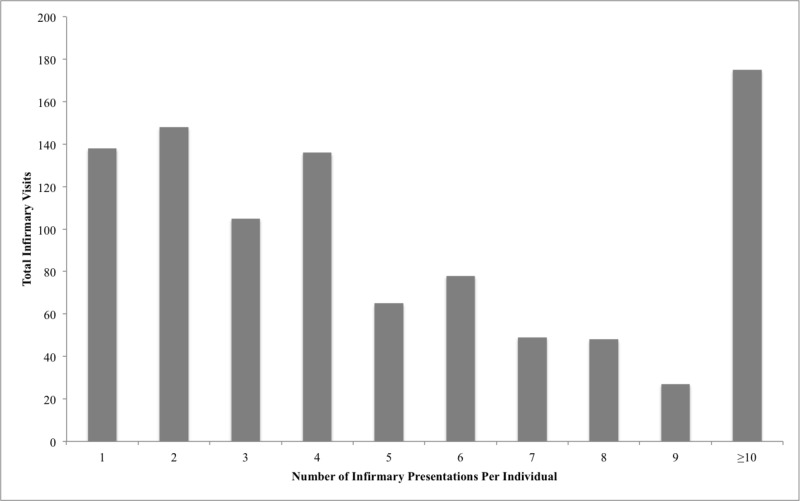
Infirmary utilization during the second summer Data presented in this figure was collected in year 2 of the study only. ID numbers were analyzed to assess repeat presentors. Total infirmary visits = number of visits to the infirmary during the summer per individual multiplied by the number of individuals who presented to the infirmary the specified number of times.

Fifty-one percent of people presenting to the infirmary had been to the infirmary in the past seven days. In the second summer, the mean infirmary presentation frequency per camp resident was 2.9 presentations. One hundred ninety-seven individuals presented more than once and one person presented more than 20 times (Figure 4).

**Figure 5:**
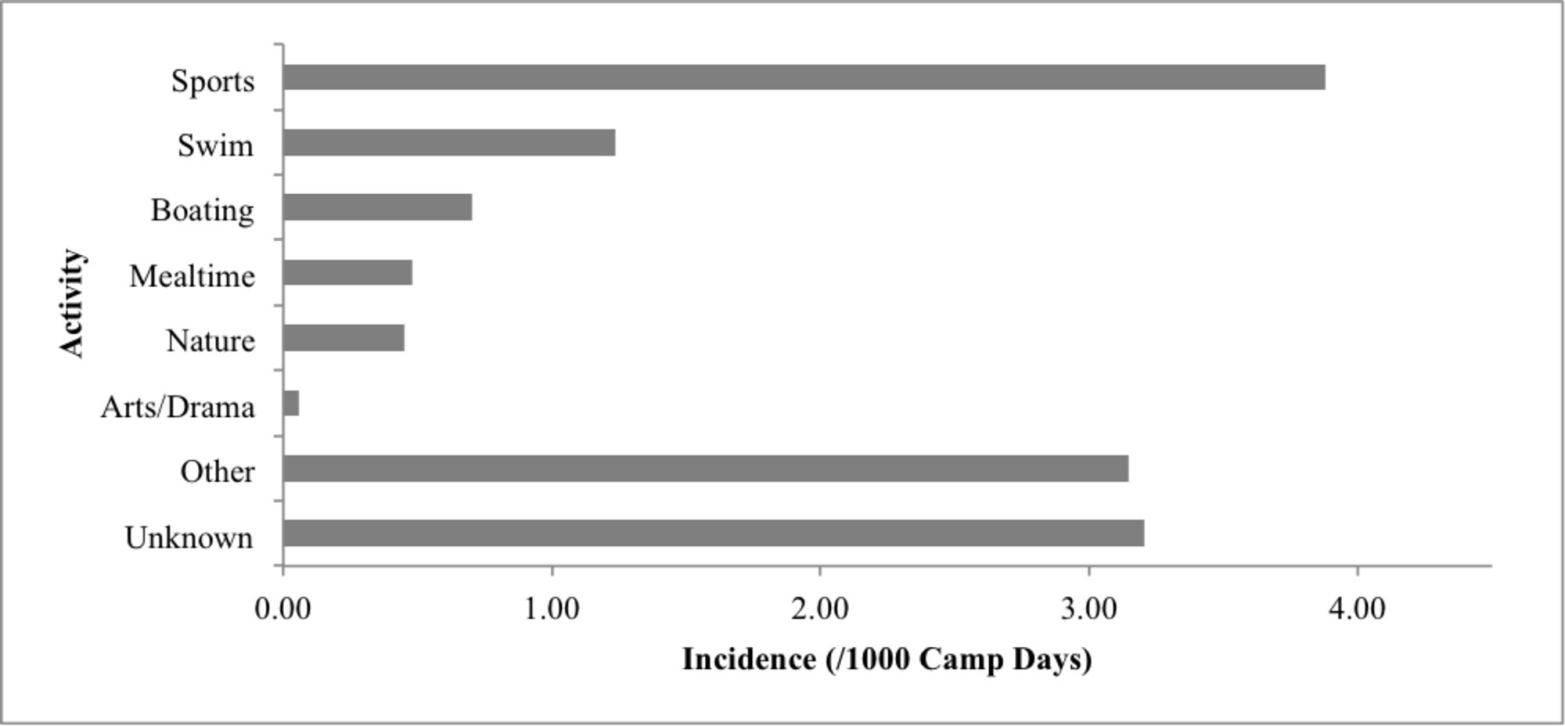
Activity at time of injury per 1000 camp days

The lower limb was the most common body region involved in injuries (44.9%), followed by upper limb (26.6%), and head/neck (15.8%) (Table 3). The most common activities at the time of injury were sports (3.9 per 1000 CD) and waterfront activities (1.9 per 1000 CD). Unstructured time, such as rest period and after curfew, as well as evening or camp-wide programs constitute the “other” category (3.2 per 1000 CD) (Figure 5). The location of injury in camp was most commonly sports courts (2.7 per 1000 CD), in/around cabins (2.5 per 1000 CD), and at the waterfront (2.0 per 1000 CD). Of the 439 injury presentations that included documentation regarding staff supervision, 69.2% of injuries occurred in the presence of staff, while 30.8% of injuries were not supervised (Table 3).

**Table 3:** Characteristics of injuries presenting to the infirmary /1000 CD = presentations per 1000 camp days. ¶ Supervision was reported in 439 injuries. The remainder were either unknown or not applicable for injuries where supervision is irrelevant such as bug bites. † Horseplay data were only collected in year 2 (2016), where there was a total of 283 injuries. * Total Injury (n) = 632

		n (%)	/1000 CD
Staff Supervision¶	Yes	304 (69)	8.5
	No	135 (31)	3.8
Horseplay Causing Injury†	Yes	45 (15.9)	1.3
	No	199 (70.3)	5.6
	Unknown	39 (13.8)	1.1
Body Part*	Lower Limb	284 (44.9)	8.0
	Upper Limb	168 (26.6)	4.7
	Head/Neck	100 (15.8)	2.8
	Trunk	28 (4.4)	0.8
	Unknown	52 (8.2)	1.5

## Discussion

This study examined the incidence of injuries and illnesses at two Canadian residential summer camps over two summers. Illnesses occurred twice as often as injuries and were most commonly communicable. Over the two years of the study, 50 individuals were sent to a hospital for further evaluation including diagnostic imaging, access to treatment that was unavailable at the camp such as casting, or assessment by a physician when one was not available on-site. None of these individuals were admitted to hospital.

The incidence of infirmary presentations in our study was higher than reported in American studies. This may be partially attributed to more frequent data collection monitoring. Additionally, our study documented every infirmary presentation rather than including only those injuries and illnesses that resulted in removal from normal camp routine for a pre-specified duration of time (e.g., more than four hours) since we wanted to accurately capture the full scope of infirmary caseload [2]. American studies with restrictive inclusion criteria found incidences of 0.47-0.5 injuries per 1000 CD and 0.93-1.38 illnesses per 1000 CD [2-3] whereas a study with comprehensive case inclusion found 67.5 infirmary presentations per 1000 CD [12]. Altogether, our findings further support the notion that injuries and illnesses at camp are generally infrequent and of low acuity [2-5].

Although senior campers had the highest absolute number of infirmary presentations, this is likely related to the higher baseline size of this age group at camp compared to junior campers and staff. Junior campers are the most frequent infirmary presenters relative to their time spent at camp. We speculate that poor hygiene may be a factor, as younger campers may be less thorough at cleaning their hands and bodies, contributing to the spread of communicable illnesses at camp [13]. Counsellors may be at higher risk of illness due to insufficient sleep, high stress, and co-habitating with their campers in sometimes unhygienic conditions. In keeping with other studies, staff had the lowest incidence of infirmary presentations despite these factors [2,7,14]. Possible explanations include staff not having time to report to the infirmary during open hours, self-medication of minor illnesses and injuries (which campers are not permitted to do), or better hand hygiene. Proper hand hygiene is known to reduce gastrointestinal and respiratory illness; thus camps should emphasize hand hygiene practice to all campers and staff [15]. 

The highest number of presentations occurred in the second week of camp, which fits with the typical length of viral incubation for common pediatric viruses such as rhinovirus, enterovirus, adenovirus, parainfluenza, and influenza [16]. There was a rapid decline in infirmary presentations during the second half of camp, which coincides with a large portion of campers leaving camp midway through the summer. The higher staff-to-camper ratio following the departure of a portion of the campers could theoretically increase supervision and possibly reduce the likelihood of illness and injury in junior campers. That said, most of the time, there was staff supervision at the time of injury. Staff supervision meant that staff was present, though not necessarily actively watching the camper, which may partly explain the high frequency of injuries caused by horseplay (3.0 per 1000 CD). Erceg et al. stressed that staff should be aware that injuries are just as likely to occur during supervised events [4].

The most common injuries were cuts/bruises, muscle/tendon injuries including sprains, and bites. As Lyme disease becomes more prevalent in Eastern North America and awareness continues to increase in the general public [17], camps will likely experience pressure to demonstrate the measures they are taking to address this issue. Both camps were located in Lyme-endemic areas and over the two summers, one camper was prescribed doxycycline due to the appearance of a “bulls-eye rash” and a known tick bite [18].

Most individuals presented to the infirmary more than once. Though children can generally be relied upon to seek care when needed [5], they may overutilize the infirmary and seek assistance for issues that in their home environment might be dealt with by a parent rather than a healthcare provider.

Limitations

This study has several limitations. Data regarding household income, family structure, and place of residence were not collected. The camps may have varied in the characteristics of the camper and staff cohorts, the activities provided by the camp and the camp facilities themselves. While our results are consistent with the findings of a 1974 American study, larger scale Canadian studies may still be needed [12]. Differences in injury and illness definitions used in more recent American studies make comparisons difficult.

In year one of this study, we did not document identification numbers. This precluded the ability to assess the effect of repeated infirmary presentations. We did not collect data as to the severity of illness/injury and cannot quantify the impact it had on the camper in terms of time lost.

As with any study that involves data collection tools administered by multiple observers, there is a risk of inter-observer variability. In order to maximize consistency in data collection, all staff responsible for entering data participated in interactive pre-camp training as well as frequent reminders and debriefing meetings. These meetings gave data collectors the opportunity to ask questions about complex or ambiguous cases in a positive, supportive setting and addressed issues that needed to be amended after year one. Our study likely captured the full range of illness presentations at camp, as treatments for camper illnesses are not accessible outside of the infirmary. However, it is possible that simple injuries were underreported, as they are often treatable by general camp staff trained in basic first aid and do not warrant a presentation to the infirmary.

## Conclusions

Overall, these summer camps provided a safe environment for children, as injuries and illnesses requiring assessment at a hospital were infrequent. Despite this, camp infirmaries were highly utilized, particularly by junior campers. Illnesses at camp occurred almost twice as frequently as injuries. Most illnesses were communicable in nature and peaked in the second week of camp. This study compares similarly to United States (US) studies that looked at presentation statistics. The results of this study could be used to inform injury and illness prevention strategies at Canadian residential summer camps. Future studies should focus on developing and implementing interventions to reduce the spread of respiratory illnesses at camps.

## Appendices

Appendix I: Sample Camp Day Calculations

1) Sample Camp Day Calculation

Incidence of Illness  = (number of illness presentations)*1000 / (number of campers * average number of days campers stayed at camp)

For example: If a camp had 500 campers over the summer, who stayed an average of 20.3 days, and 45 cases of sore throat (ST) were reported over the summer:

Incidence of ST         = 45 cases of ST *1000 / (500 campers * 20.3 days/camper)

                                    = 4.43 / 1000 camp days

2) Sample Age-Specific Camp Day Calculation

Age Specific Incidence of Illness    = (number of illness presentations)*1000 / (number of campers * average number of days campers within 1 age group stayed at camp)

For example: A camp had 500 campers, and had 50 staff, 150 senior campers and 300 junior campers over the summer. The junior campers stayed at camp on average for 20 days and the senior campers and staff stayed on average for 30 days. There were 45 cases of sore throat (ST) were reported over the summer (Jr - 15, Sr - 20, Staff - 10):

Age Specific Incidence of ST

Jr campers

 = 15 cases of ST *1000 / (300 campers * 20 days/camper)

= 4.43 / 1000 camp days

Sr campers

 = 20 cases of ST *1000 / (150 campers * 40 days/camper)

= 4.43 / 1000 camp days

Staff

 = 10 cases of ST *1000 / (50 campers * 40 days/camper)

= 4.43 / 1000 camp days
